# Ultrastructural Profile Combined with Immunohistochemistry of a Hepatic Progenitor Cell Line in Pediatric Autoimmune Hepatitis: New Insights into the Morphological Pattern of the Disease

**DOI:** 10.3390/cells10081899

**Published:** 2021-07-27

**Authors:** Joanna Maria Lotowska, Maria Elzbieta Sobaniec-Lotowska, Piotr Sobaniec

**Affiliations:** 1 Department of Medical Pathomorphology, Faculty of Medicine with the Division of Dentistry and Division of Medical Education in English, Medical University of Bialystok, 15-269 Bialystok, Poland; joanna.lotowska@umb.edu.pl; 2 Department of Pediatric Neurology and Rehabilitation, Faculty of Health Sciences, Medical University of Bialystok, 15-274 Bialystok, Poland; piotr.sobaniec@neuromaster.pl

**Keywords:** pediatric autoimmune hepatitis (AIH), hepatic progenitor cell line (HPC line), ultrastructural types of HPCs, hyperactive Kupffer cells, transitional hepatic stellate cells (T-HSCs), liver fibrogenesis, transmission electron microscopy (TEM), immunohistochemistry for CK7

## Abstract

Considering that the heterogenic population of a hepatic progenitor cell line (HPCL) can play a vital role in autoimmune hepatitis (AIH), we decided to conduct pioneering retrospective evaluation of these cells in pediatric AIH by means of transmission electron microscopy (TEM). The aim of the study was to assess the ultrastructure of the HPCL in children with untreated AIH. Ultrastructural analysis of the HPCL population, preceded by immunohistochemical staining for cytokeratin 7 (CK7), was performed using pretreatment liver biopsies from 23 children with clinicopathologically diagnosed AIH. Immunohistochemical assessment for CK7 allowed detection of proliferating immature epithelial cells differentiating towards periportal and intralobular intermediate hepatocytes without marked formation of ductular reactions in AIH children. Using TEM, we distinguished three morphological types of HPCs: I—the most undifferentiated progenitor cells; III—intermediate hepatocyte-like cells; II—intermediate bile duct cells. Most frequent were the cells differentiating towards hepatocytes, most rare—those differentiating towards cholangiocytes. The results indicate that an HPCL may be an important source of hepatocyte regeneration. Ultrastructural analyses of the HPCL population, combined with immunohistochemistry for CK7, might be a useful tool to evaluate liver cell regeneration, including fibrogenesis, and may help better understand the morphological pattern of the disease, in pediatric AIH. Frequent appearance of an HPCL in the vicinity of fibrotic foci, often accompanied by hyperactive Kupffer cells and transitional hepatic stellate cells, may indicate their significant involvement in liver fibrogenesis.

## 1. Introduction

Autoimmune hepatitis (AIH) is a poorly known immune-mediated autodestructive acute or chronic liver disease with female predominance and potential for the development of liver fibrosis [1,2,3,4,5,6,7,8]. AIH is characterized by elevated serum aminotransferase levels, the presence of hypergammaglobulinemia, circulating autoantibodies, and interface hepatitis with portal plasma cell lymphocytic necroinfiltration and rosetting of hepatocytes in histological examination [3,4,5,6,7,8,9,10,11]. Interface hepatitis in this pathology is closely related to the process of liver fibrosis [1,4,5,10,11,12,13]. It is a relatively rare but devastating disease, particularly aggressive in children/adolescents, leading in some cases to cirrhosis, liver failure, and death [5,7,10,12,13,14,15].

It should be emphasized that pathomorphological examination of liver biopsy still remains the criterion standard in both initial diagnosis and long-term follow-up of patients with AIH, especially before the start of immunosuppressive therapy [1,3,4,5,16,17,18]. Early histological diagnosis extended with immunohistochemistry (IHC) is indispensable for a better outcome [5,16,17,18,19]. However, the diagnostic analysis of AIH by means of transmission electron microscopy (TEM), which facilitates histopathological assessment of the pathology and its early diagnosis, particularly in diagnostically difficult cases, is too seldom applied [4].

Importantly, using TEM, we recently identified a novel ultrastructural feature for the diagnosis of childhood AIH, i.e., characteristic glassy droplet inclusions within the cytoplasm of marked activated Kupffer cells/macrophages (KCs/MPs) [4]. We were also the first in the hepatological literature to provide a detailed TEM assessment of an interesting sequence of morphological lesions in liver sinusoidal endothelial cells, markedly involved in the morphogenesis and progression of AIH, coexisting with abnormalities in KCs/MPs [11]. An increasing number of interesting immunohistochemical reports has lately appeared, characterizing the picture of the heterogenic population of small immature epithelial cells of the hepatic progenitor cell line (HPCL) nature in adult patients with AIH and their role in disease progression [5,17,18,19,20]. However, no such reports have been available on pediatric AIH. Moreover, we failed to find studies concerning the ultrastructural identification of the HPCL that would involve the respective types of these cells in patients with AIH, both adults and children.

The above data, especially the immunohistochemical evaluations of HPCL using cytokeratin 7 (CK7) in adult patients with AIH by Fujiwara et al. [5,17,18] and Verdonket al. [19] inspired us to undertake microscopic investigations of HPCL in this pathology in children.

The HPC line, collectively termed oval cells in rodents, and liver progenitor cells (LPCs), oval-like progenitor cells, or liver stem cells in patients, includes very small immature epithelial cells that reside in the smallest ramifications of the biliary tree. They form the heterogenic cell population and in a healthy liver account for only 1–3% of the normal liver cell pool, located mainly in the portal and periportal area [20,21,22,23,24,25,26,27]. It has been shown in both the animal and human liver that HPCLs have a bipotent nature, i.e., exhibit a two-directional differentiating ability to differentiate towards the biliary and hepatocyte lineages. It is assumed that these cells constitute a major source of precursor cells both for hepatocytes and epithelial cells of bile ductules, and are therefore defined in the literature as hepatic parenchymal cells with intermediate (hepatocyte/biliary) features [27,28,29,30,31,32,33,34]. Recently, by lineage tracing using the Wnt-responsive gene Axin2 in mice, Wang et al. identified a population of other liver progenitor cells, i.e., of proliferating and self-renewing cells adjacent to the central vein in the liver lobule [35]. It is assumed that these pericentral stem/progenitor cells are responsible for maintaining homeostasis in the uninjured liver. The diversity and plasticity of these HPCs and their anatomical niches are also reported, and their important roles in liver regeneration, fibrosis, and cancers are discussed [35,36].

Taking the above into account, especially the lack of morphological reports concerning the heterogenic population of an HPCL in pediatric patients with AIH, we aimed to assess this cell line in children with untreated AIH using ultrastructural analysis by means of TEM. To better evaluate the morphological nature of pediatric AIH, our submicroscopic observations of the HPC line were preceded by immunohistochemistry with CK7, one of the most specific and earliest markers of liver progenitor cells, especially of intermediate hepatocytes.

From the pathomorphological point of view, particularly interesting is the ultrastructural identification of the respective types of cells in the assessed population and the submicroscopic picture of their closest surroundings, never before conducted in any, even adult patients with AIH.

The current study is a continuation of our earlier ultrastructural research conducted by means of TEM in pediatric AIH [4,11] and also of similar submicroscopic observations of the population of LPCs in other chronic liver diseases in children [37,38,39,40] and in the experimental model of biliary fibrosis [25].

## 2. Materials and Methods

### 2.1. Study Patients’ Profile

Retrospective immunohistochemical and ultrastructural investigations were conducted using tissue material embedded in paraffin and epon blocks, including pretreatment needle liver biopsy specimens obtained from 23 children (7 boys and 16 girls) aged 4–17, hospitalized at the Department of Pediatrics, Gastroenterology, Hepatology, Nutrition, and Allergology, Medical University of Bialystok, with clinicopathologically diagnosed AIH.

The clinical data, including immunological and serological disturbances in blood serum and differential diagnostics of patients were reported in our earlier papers [4,11].

The collected material was subjected to pathomorphological, i.e., histological, histochemical, immunohistochemical and ultrastructural analyses using TEM at the Department of Medical Pathomorphology, Medical University of Bialystok.

Hepatic necroinflammatory injuries and fibrosis had been previously assessed histologically using routine Mayer’s hematoxylin and eosin (H&E) staining. Additionally, liver fibrosis (stained for collagen fibers and reticulin fibers) was determined by a panel of histochemical stains and assessed by a single hepatopathologist blinded to patient clinical data.

The study showed typical histological features of AIH, i.e., interface and lobular hepatitis, moderate/severe in nature, with portal infiltration of lymphocytes and plasma cells, severe necroinflammatory reaction, and rosette formation of hepatocytes. The alterations were frequently accompanied by portal, periportal, and bridging fibrosis.

The stage of fibrosis (staging—S; range: 0–4) in liver biopsies was retrospectively scored using the semiquantitative scoring system according to Batts and Ludwig [41], which was in some cases complemented by the scoring system proposed by Ishak et al. [42]. In the group of 23 children, we identified four patients with advanced liver fibrosis (corresponding to S3) but not with liver cirrhosis, seven—with mild (S1) liver fibrosis, 12—with moderate (S2) liver fibrosis.

Ultrastructural identification of different cell types of the HPCL was based on the report by Roskams et al. [43].

We would like to emphasize that for obvious bioethical as well as procedural reasons, we could not have control groups consisting of healthy children subjected to liver biopsy as liver biopsy, being an invasive procedure, is not performed in healthy children. This is clearly stated by the ESPGHAN Hepatology Committee in the 2015 guidelines for pediatric liver biopsy [44]. Therefore, describing the ultrastructure of the HPC line in pediatric AIH, we compared it to the same cell population but in other chronic liver diseases in children investigated at our center, namely chronic hepatitis B [37,38] and nonalcoholic steatohepatitis (NASH) [40].

Informed consent was obtained from parents of each patient included in the study. The current research was approved by the Bioethics Committee, Medical University of Bialystok.

### 2.2. Liver Tissue Processing for Immunohistochemistry for Cytokeratin 7 (CK7)

This study was conducted routinely using paraffin-embedded liver biopsy specimens by means of a standard detection system EnVision FLEX K8002 (Dako) and anti-CK7 (FLEX Monoclonal Mouse Anti-Human Cytokeratin 7, Clone OV-TL 12/30, Ready-to-Use; Dako).

The application of immunohistochemical staining for CK7 allowed visualization of the population of the HPCL, the bile duct, and the ductular epithelium.

### 2.3. Liver Tissue Processing for Transmission Electron Microscopy

For TEM, fresh small tissue blocks (1 mm^3^ volume) from the liver biopsy material were primarily fixed in Karnovsky fixative (containing 2% paraformaldehyde and 2.5% glutaraldehyde in 0.1 M cacodylate buffer, pH 7.4) for 12 h. Then, the specimens were post-fixed in 2% osmium tetroxide (OsO_4_) in 0.1 M cacodylate buffer (pH 7.4) for 1 h. Subsequently, the material was dehydrated through a graded series of ethanols and propylene oxide, embedded in Epon 812 or Gycid ether 100 for electron microscopy, and sectioned on a Reichert ultramicrotome (Reichert Ultracut S) to obtain semithin sections, which were stained with 1% methylene blue in 1% sodium borate. Ultrathin sections (approximately 70 nm) were cut with the same Reichert ultramicrotome, mounted on copper grids, contrasted with uranyl acetate and lead citrate, and then examined using an Opton EM 900 electron microscope (Oberkochen, Germany) and photographed with a TRS camera (CCD camera for TEM 2K inside). This processing procedure had been used in our earlier ultrastructural investigations of the liver in children [4,11,45]. The HPC line was determined by a microscopist who was blinded to the clinical information.

## 3. Results

### 3.1. Immunohistochemical Staining for CK7

**Figure 1:**
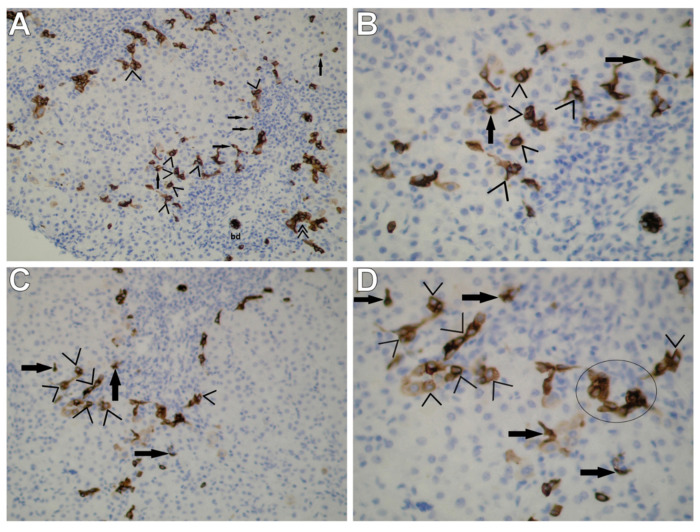
Immunohistochemical staining for CK7 in liver biopsies obtained from children with AIH shows immunoreactive cells of the HPC line in periportal and intralobular regions; density inflammation is observed in portal tracts. (**A**) Periportal and intralobular CK7-positive cells—intermediate hepatocytes (pIMs and iIMs (>)) corresponding to IHCs observed by means of TEM are well-visible, especially in the center of the photograph (on the border between the periportal region and the limiting plate); focally scarce, dispersed, tiny periportal and intralobular progenitor cells (pPCs and iPCs (→)). At the periphery of portal tracts (i.e., at the photograph’s margins), a poor ductular reaction (DR (>>)) at the portal–parenchymal interface was observed—very few neoforming bile ductules as a result of hepatic progenitor cell activation with no or very poorly defined lamina, not surrounded by a continuous basement membrane. A mild DR is accompanied by massive inflammatory infiltration; bd—bile ductule. (**B**) Greater magnification of the central fragment of Figure 1A visualizes numerous CK7-positive cells of the nature of iIMs (>) penetrating the hepatic lobule well; present are also single dispersed CK7-positive cells of the nature of PCs (→)—very small cells with an oval nucleus and a small rim of the cytoplasm (corresponding to UPCs observed by means of TEM). PCs exhibit a strong homogenous cytoplasmic and membranous staining pattern; on the other hand, iMs—intermediate in size—demonstrate a variable cytoplasmic and membranous staining pattern; S1 (**A**,**B**). Original magnification: ×10, scale bar: 200 µm (**A**); ×40, scale bar: 50 µm (**B**). (**C**) Immunohistochemistry for CK7 demonstrates the picture of numerous periportal intermediate hepatocytes (pIMs (>)) and few periportal and intralobular hepatic progenitor cells (pPCs and iPCs (→)). Portal tract—dilated, with dense inflammation. (**D**) Greater magnification of the central fragment of Figure 1C clearly demonstrates the immunohistochemical pattern of intermediate hepatocytes (>), mainly located in the periportal region; rare immunoreactive progenitor cells present in the vicinity (→). In the “circle,” the microscopic picture may indicate neoformation of a bile ductule, still without its own lumen or basement membrane; S2 (**C**,**D**). Original magnification: ×20, scale bar: 100 µm (**C**); ×40, scale bar: 50 µm (**D**).

In all the patients with AIH, immunohistochemical staining for cytokeratin 7 showed a commonly present positive or strongly positive population of small/very small and immature epithelial cells in periportal and intralobular regions corresponding to the HPCL (Figure 1A–D). However, ductular reactions (DRs) were by far less common in our patients (Figure 1A).

The population of the immunoreactive HPCL included numerous cells differentiating towards hepatocytes defined as intermediate hepatocytes (IMs) (corresponding to type III cells observed by means of TEM) and also, less often, as immature progenitor cells (PCs) (corresponding to type I cells observed by means of TEM), many a time dispersed among IMs that were bigger.

Taking into account the location of intermediate hepatocytes, periportal IMs (pIMs) and intralobular IMs (iIMs) were distinguished. Since PCs showed a similar location to that of IMs, they were defined as periportal PCs (pPCs) and intralobular PCs (iPCs) (Figure 1A–C).

PCs appeared as very tiny CK7-positive cells with an oval nucleus and a small rim of the cytoplasm. These cells demonstrated a strong homogenous cytoplasmic and membranous staining pattern located distally to DRs (Figure 1B,D). On the other hand, intermediate hepatocytes were seen as cells intermediate in size and with an immunohistochemical staining pattern for CK7 between that of hepatic progenitor cells and hepatocytes. They showed a variable cytoplasmic and membranous staining pattern. IMs were mainly located in the periportal areas, occasionally penetrating the lobular areas (Figure 1B,D).

Interestingly, in the vast majority of AIH patients, no distinct ductular reactions were observed. Only in four cases (17.4%) of AIH, moderate proliferation of bile ductules was found at the periphery of portal tracts.

### 3.2. Transmission Electron Microscopic Analysis

We performed comparative analysis of liver ultrastructure of the HPCL in children with AIH versus children with a non-AIH disease in the same age group with reference to our previous electron microscopic research into chronic liver diseases, namely chronic hepatitis B [37,38] and nonalcoholic steatohepatitis [40].

Our ultrastructural study showed a quite prominent number of cells corresponding to the population of the HPCL in all the study children with clinicopathological diagnosis of AIH. Interestingly, the cells occurred in greatest numbers in patients with coexisting advanced and moderate liver fibrosis (the total of 17 patients) where bundles of collagen fibers were seen to adhere directly to these cells or were situated in their vicinity. They were encountered in various parts of the hepatic lobule, but mostly in the periportal area in the vicinity of the limiting plate of the lobule.

**Figure 2:**
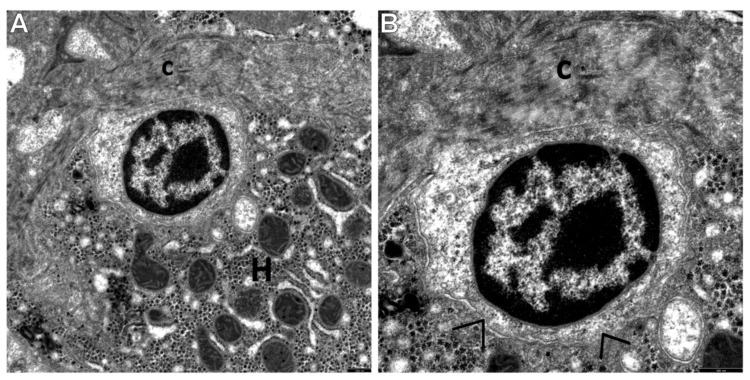
Electron micrographs showing a centrally located, very primitive-looking undifferentiated hepatic progenitor cell (UPC) of type I in a liver biopsy obtained from a child with AIH. (**A**) The UPC does not show any signs of differentiation. It is very small in size, oval in shape, has scant cytoplasm, markedly high nucleus/cytoplasm ratio, and undifferentiated cytoplasmic structures. The nucleus of this cell is large, round, and contains dense heterochromatin clumped under the nuclear envelope and well-pronounced small nuclear bodies surrounded by the euchromatin band. H—hepatocyte. A thick bundle of collagen fibers (c) surrounds the progenitor cell from the top and adheres closely to it. Scale bar: 1 µm. (**B**) Higher magnification of the UPC well demonstrates an almost complete lack of cell organelles within a relatively light cytoplasm with microgranular structure; above the cell, adjacent collagen fibers (c) are well seen to accumulate. Between the UPC and the adjacent mature hepatocyte, point desmosomes (intercellular junctions) (>) are present. Scale bar: 0.5 µm.

**Figure 3:**
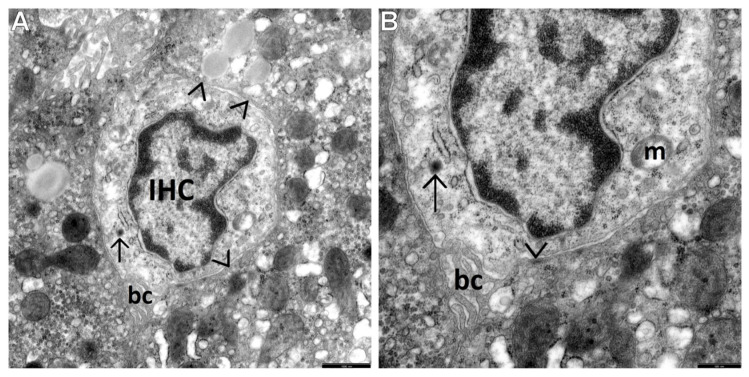
The view of a small (although bigger than the UPC) oval progenitor cell, markedly differentiating towards the hepatocyte lineage, i.e., an intermediate hepatocyte-like cell (IHC) in a liver biopsy obtained from a child with AIH. (**A**) IHC contains primitive-looking cytoplasmic structures in the electron-light cytoplasm (more numerous, however, as compared to the UPC in Figure 2). The cell organelles (better visualized in Figure 3B) include single mitochondria, very short channels of the granular endoplasmic reticulum, a small round structure that could correspond to a newly formed peroxisome (→) and glycogen rosettes; hemicanaliculus (i.e., an almost formed biliary canaliculus) is clearly seen—bc. The cell shows a high nucleus/cytoplasm ratio and a marginal location of heterochromatin. Hepatocytes that surround the IHC show features of acinar proliferation of the smooth endoplasmic reticulum. Point desmosomes (>) between the IHC and the adjacent hepatocyte. Scale bar: 1 µm. (**B**) Higher magnification of the IHC well demonstrates the picture of primitive organelles, including a mitochondrion (m) and an already nearly formed biliary canaliculus (bc) located at the bottom pole of the cell. Scale bar: 0.5 µm.

**Figure 4:**
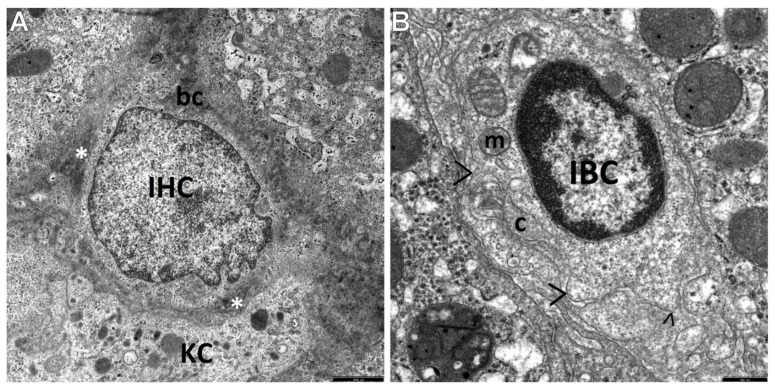
The view of two cells present in the intercellular space (between hepatocytes)—a primitive-looking cell of the HPCL differentiating towards the intermediate hepatocyte-like cell (IHC) and the oval cell differentiating towards the biliary lineage, i.e., the intermediate bile-like cell (IBC) in a liver biopsy obtained from a child with AIH. (**A**) The cell nucleus of the IHC being formed fills up the cell (the nucleus/cytoplasm ratio is evidently shifted for the nucleus), with reduced electron density of chromatin. The cell cytoplasm, forming a very narrow rim around the cell nucleus, filled up with microgranular material, containing the minimum amount of primitive cell organelles; at the upper pole of the cell, a contour of a structure likely to correspond to a very primitive biliary canaliculus—hemicanaliculus (bc). Hyperactive Kupffer cell/macrophage (KC) containing numerous phagosomes adheres to its bottom pole. Around the IHC and the KC, the presence of a flocculent, condensed extracellular matrix (*), which can be referred to as the morphological precursor of collagen. Scale bar: 1 µm. (**B**) Within the IBC cytoplasm, the presence of rare primitive-looking organelles, mainly mitochondria and elements of the smooth endoplasmic reticulum; the nucleus/cytoplasm ratio markedly shifted for the cell nucleus. The cell has well-developed plasma membrane filopodia (>), well-developed basal membrane and pinocytotic vacuoles; visible is the transverse section through the bundle of mature collagen fibers (c) closely adhering to the cell. Scale bar: 0.5 µm.

The current investigations allowed us to distinguish three main types of cells among the population of the HPCL, including type I cells (the most undifferentiated hepatic progenitor cells—UPCs (Figure 2A,B)) and type III cells (the most frequently encountered intermediate hepatocyte-like cells—IHCs (Figure 3A,B and Figure 4A)).

**Figure 5:**
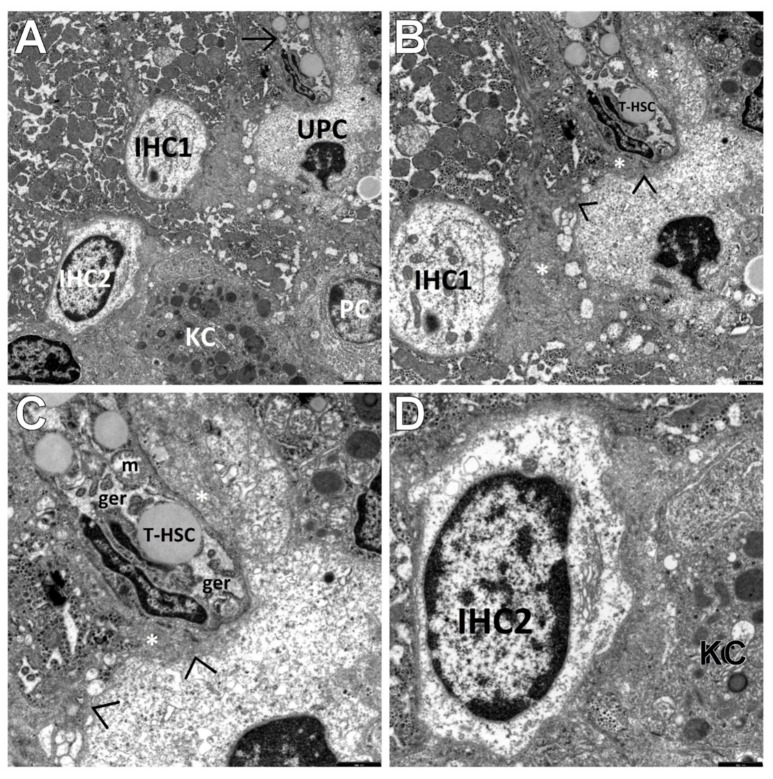
Very interesting electron micrographs demonstrating the ultrastructure of various types of liver progenitor cells and NPCs adhering to them in a liver biopsy obtained from a child with AIH. (**A**) The view of three cells of the HPCL present in the periportal area; among them, one of the most primitive-looking hepatic progenitor cells (UPC) and two more differentiated cells corresponding to the intermediate hepatocyte-like cells (IHC1 and IHC2). They are small in size, oval in shape, with electron-light cytoplasm. The UPC has undifferentiated cytoplasmic structures, whereas IHC1 and IHC2 have both primitive and relatively well-developed cell organelles, mainly mitochondria (in IHC1) and elements of the endoplasmic reticulum (in IHC1 and IHC2), in which channels of the granular endoplasmic reticulum prevail. The nucleus of IHC2 is large, oval, much less abundant in heterochromatin compared to the UPC nucleus, with a marginal location of heterochromatin. A transitional form of the hepatic stellate cell (T-HSC) (→) closely adheres to the top pole of the UPC, squeezing into it in places and deforming its shape. At the bottom of the electronogram, there are a fragment of the hyperactive Kupffer cell/macrophage (KC) and a fragment of the plasma cell (PC). Scale bar: 2.5 µm. (**B**,**C**) Greater magnification well demonstrates the morphological picture of the UPC and the adjacent cells; visible is a direct contact of the UPC via junctional complexes (>) with the T-HSC and the hepatocytes. The T-HSC is enclosed by flocculent material of increased electron density (*) that could correspond to the morphological precursor of collagen fibers. The T-HSC lost a considerable part of lipid material and contains a markedly dilated granular endoplasmic reticulum (ger) and a swollen mitochondrion (m). Scale bar: 1 µm (**B**); 0.5 µm (**C**). (**D**) Greater magnification of the IHC2 shows relatively poorly developed organelles accumulated at the nucleus within a very light cytoplasm, i.e., elements of the endoplasmic reticulum with the Golgi apparatus being formed, tiny vacuolar structures and glycogen rosettes. In a very close vicinity of the IHC2, a fragment of the hyperactive KC can be seen. Scale bar: 0.5 µm. In all the above panels: osmium tetroxide/uranyl acetate/lead citrate.

However, type II cells, i.e., intermediate bile duct-like cells (IBCs (Figure 4B)), were relatively rare in our study. HPCL-type cells were seen single or in clusters of two (usually of the same type—UPCs or IHCs), seldom in groups of three or four (Figure 5A).

Type I cells, i.e., UPCs, were the most primitive-looking in the population of the HPCL. They were very small, oval or round in shape. Usually, their diameter did not exceed 5 microns. Their large nucleus contained dense and highly clumped heterochromatin which accumulated markedly under the nuclear envelope, as well as euchromatin which was less abundant. The cytoplasm of UPCs was relatively scarce and brighter than in the surrounding mature hepatocytes (Figure 2A,B). In consequence, the nucleus-to-the-cytoplasm ratio was very high. Worthy of note is the fact that the number of cytoplasmic structures was very small, and they were only minimally differentiated (Figure 2A,B and Figure 5A–C). Some UPCs had intercellular junctions (point desmosomes), which linked them to the adjacent fully mature hepatocytes (Figure 2B) or to the adjacent nonparenchymal hepatic cells (NPCs) (Figure 5B,C).

Type III cells, i.e., IHCs, were bigger than type I cells (maximally nearly twice as large as UPCs), although their diameter did not exceed one half of the diameter of the mature hepatocytes, usually being smaller than 9 microns. Their nuclei were large, predominantly oval in shape, less abundant in heterochromatin compared to the UPC nuclei, and often resembled the nuclei of mature hepatocytes. The cytoplasm of IHCs was bright—it showed low electron density and contained better developed cell organelles compared to those of UPCs. These were mainly mitochondria and elements of the endoplasmic reticulum, with the predominance of channels of the granular endoplasmic reticulum (ger). Among the intracellular organelles, there were also glycogen-associated er complexes, small structures that could correspond to newly formed peroxisomes, and sometimes the Golgi apparatus (Figure 3A,B and Figure 5A,B,D). The organelles were accumulated in the vicinity of the nuclear poles (Figure 5A,D) or irregularly scattered throughout the cytoplasm (Figure 3A,B). Many times such cells showed on their secretion pole newly formed bile canaliculi (Figure 3A,B and Figure 4A). Some IHCs, although less frequently than UPCs, had point desmosomes connecting them to the adjacent fully mature hepatocytes or NPCs (Figure 3A,B).

On the other hand, type II cells, i.e., IBCs, the least common subpopulation of the HPCL studied, were mainly found within some segments of the biliary system in the neighborhood of mature cholangiocytes. IBCs were usually approximately 10 microns in size and had features characteristic of cholangiocytes. As compared to UPCs, they had increased numbers of cytoplasmic organelles and well-developed plasma membrane filopodia (Figure 4B).

In the population of the HPCL, we sometimes observed stem cells showing features ranging from marked destruction to total disintegration accompanied by damaged hepatocytes.

It should be noted that in the dilated spaces of Disse or in their vicinity, activated nonparenchymal hepatic cells (NPCs), i.e., Kupffer cells/macrophages and hepatic stellate cells, including the transitional form of these cells (T-HSCs), were often found to adhere to the population of the HPCL (Figure 4A and Figure 5A–D). The T-HSCs, being morphologically involved in fibrogenesis, appeared as elongated cells that had lost a considerable part or almost all lipid material and contained well-developed, markedly dilated channels of the ger (Figure 5A–C) and the Golgi apparatus. Various types of liver progenitor cells were frequently accompanied by flocculent material that can be referred to as the morphological precursor of collagen fibers (Figure 4A and Figure 5B,C) and mature collagen fiber bundles, directly adhering to these cells (Figure 2A,B and Figure 4B) or lying within a short distance from them.

## 4. Discussion

It is currently accepted that TEM and/or immunohistochemistry with a panel of antibodies against markers of the HPCL (c-kit, Ov6, CD34, CD56, CK7, CK8, CK18, CK19, and chromogranin A) and their progeny are necessary to detect these cell types, which are very small in size and very few, hardly recognizable with H&E or other routine staining [20,22,23,24,25,26,27]. The immunohistochemistry staining for cytokeratin 7 used in our study allowed us to detect the expansion of two subpopulations of immunoreactive cells in liver biopsies of all children with AIH without distinct formation of ductular reactions. These were intermediate hepatocytes (corresponding to IHCs observed by means of TEM), classified by location as periportal and intralobular IMs and less numerous and finer progenitor cells, i.e., PCs (corresponding to UPCs observed by means of TEM). PCs were very small in size and demonstrated a strong homogenous cytoplasmic and membranous staining pattern. On the other hand, the subpopulation of IMs consisted of cells intermediate in size and with an immunohistochemistry pattern for CK7 between that of PCs and hepatocytes.

Interesting were the results of morphological investigations of the proliferating HPCL in pediatric AIH conducted by means of TEM, constituting the main aim of our study. We were the first to distinguish and characterize the respective morphological types of this cell population, namely type I cells—UPCs, i.e., the most undifferentiated cells, type III cells—IHCs, i.e., the cells differentiating towards hepatocytes, and type II cells—IBCs, i.e., the cells differentiating towards cholangiocytes. The most common in this pathology were IHCs, as compared to IBCs that were the least common. The submicroscopic assessment revealed that UPCs were characterized by the smallest size in the population of the HPCL, had a large nucleus and a very small number of minimally differentiated cytoplasmic structures. In turn, IHCs were bigger than the most primitive-looking UPCs and contained better developed cell organelles, mainly mitochondria and elements of the endoplasmic reticulum. Many a time their secretion pole showed a developing biliary canaliculus.

The expansion of the HPCL, both of type I cells and type III cells, in the vicinity of developing or already formed fibrotic foci has been relatively frequently observed in ultrastructural investigations, which may indicate their significant involvement in fibrogenesis, especially in the initiation of this process. These cells were frequently accompanied by activated NPCs, i.e., hyperactive KCs/MPs and T-HSCs. Frequent appearance of the HPCL in the vicinity of fibrotic foci, often accompanied by hyperactive Kupffer cells and transitional hepatic stellate cells, may indicate their significant involvement in liver fibrogenesis. Worthy of note is that the current findings of submicroscopic investigations of LPCs are nonspecific and very close to our previous results obtained in pediatric patients with progressive liver fibrosis in the course of chronic hepatitis B [37,38] and nonalcoholic steatohepatitis (NASH) [39,40].

Reports of some authors as well as our own studies have revealed that the increased population of the HPCL referred to as their “activation” and differentiation towards hepatocytes or bile duct epithelial cells, or both, is a component of numerous human chronic liver diseases. The extent of progenitor cell immunoreaction and the direction of differentiation are correlated with the severity of the disease and the type of the mature epithelial cell, i.e., the hepatocyte or the bile duct epithelial cell, respectively, that is damaged [20,28,29,30,31,32].

As shown by our morphological studies of liver biopsies from children with AIH, the application of immunohistochemistry for CK7 extended with submicroscopic assessment by means of TEM allows identification of the HPC line. The observation of progenitor cell proliferation, especially of the cells differentiating towards hepatocytes, without marked formation of DRs suggests that the properties of liver regeneration in the vast majority of our patients with AIH (82.6%) were not impaired. Fujiwara et al. reported similar immunohistochemical findings for CK7 in non-severe and recovered AIH in adult patients [5,17], whereas El-Araby et al.—in children with chronic hepatitis C [31].

It should be assumed that progenitor cell expansion, like in other chronic liver diseases [20,28,29,30,31,32], can be an important source of hepatocyte regeneration in pediatric AIH as well.

Worthy of note is that the current ultrastructural assessment may also indicate a significant relationship between the expansion of the HPCL and promoting the process of hepatic fibrosis in children with AIH. This relationship is consistent with our hypothesis and the assumptions of other authors that this cell population could send signals to NPCs, especially to hepatic stellate cells, thus increasing the risk of fibrogenesis in chronic liver diseases [25,46,47,48]. This was suggested by our electron microscopic examinations of the HPC line in children with chronic viral hepatitis [37,38] and NASH [39,40] and also in the experimental secondary model of liver fibrosis in young rats submitted to common bile duct ligation [25]. However, the thorough clarification of a very complex correlation between the expansion of the HPC line and collagen fibroplasia of the liver in pediatric AIH requires further in-depth multicenter observations.

## 5. Conclusions

The current ultrastructural analysis of the HPCL population by means of TEM combined with the immunohistochemical study of this cell line using CK7 might be a useful tool for evaluating liver cell regeneration that involves the process of fibrogenesis in pediatric patients with AIH. These morphological observations indicate that the expansion of type III and type I cells of the HPC line, defined subsequently as intermediate hepatocyte-like cells and undifferentiated progenitor cells, may be an essential source of hepatocyte regeneration. The study results suggest that liver regeneration from the periportal HPCL to mature hepatocytes was not impaired in the vast majority of our children with this pathology. Interestingly, frequent appearance of liver progenitor cells in the vicinity of fibrotic foci, many a time accompanied by activated NPCs, i.e., KCs/MPs and T-HSCs, may indicate significant involvement of this cell line in fibrogenesis, especially in its initiation. We believe that the analysis of the ultrastructural profile of the respective types of the HPC line and the NPCs surrounding them may throw more light on the role of these cells in the dynamic nature of liver fibrosis in the course of pediatric AIH.

It should be assumed that the results obtained in children with AIH for the first time will help better understand the extremely complex pathomorphological pattern of the disease and might be valuable for its diagnosis.

## Data Availability

Not applicable.
